# Association of hospital volume with survival but not with postoperative mortality in glioblastoma patients in Belgium

**DOI:** 10.1007/s11060-024-04776-2

**Published:** 2024-08-02

**Authors:** Dimitri Vanhauwaert, Geert Silversmit, Katrijn Vanschoenbeek, Gregory Coucke, Dario Di Perri, Paul M. Clement, Raf Sciot, Steven De Vleeschouwer, Tom Boterberg, Cindy De Gendt

**Affiliations:** 1 Department of Neurosurgery, AZ Delta hospital Roeselare, Roeselare, Belgium; 2 Belgian Cancer Registry, Brussels, Belgium; 3 Department of Radiation Oncology, Cliniques Universitaires Saint-Luc, Brussels, Belgium; 4 Department of Medical Oncology, UZ Leuven, Leuven, Belgium; 5 Department of Oncology and Leuven Cancer Institute, KU Leuven, Leuven, Belgium; 6 Department of Pathology, UZ Leuven and KU Leuven, Leuven, Belgium; 7 Department of Neurosurgery, UZ Leuven, Leuven, Belgium; 8 Department of Neurosciences and Leuven Brain Institute (LBI), KU Leuven, Leuven, Belgium; 9 Department of Radiation Oncology, Ghent University Hospital, Ghent, Belgium

**Keywords:** Glioblastoma, Survival, Mortality, Biopsy, Outcome

## Abstract

**Objectives:**

Standard of care treatment for glioblastoma (GBM) involves surgical resection followed by chemoradiotherapy. However, variations in treatment decisions and outcomes exist across hospitals and physicians. In Belgium, where oncological care is dispersed, the impact of hospital volume on GBM outcomes remains unexplored. This nationwide study aims to analyse interhospital variability in 30-day postoperative mortality and 1-/2-year survival for GBM patients.

**Methods:**

Data collected from the Belgian Cancer Registry, identified GBM patients diagnosed between 2016 and 2019. Surgical resection and biopsy cases were identified, and hospital case load was determined. Associations between hospital volume and mortality and survival probabilities were analysed, considering patient characteristics. Statistical analysis included logistic regression for mortality and Cox proportional hazard models for survival.

**Results:**

A total of 2269 GBM patients were identified (1665 underwent resection, 662 underwent only biopsy). Thirty-day mortality rates post-resection/post-biopsy were 5.1%/11.9% (target < 3%/<5%). Rates were higher in elderly patients and those with worse WHO-performance scores. No significant difference was found based on hospital case load. Survival probabilities at 1/2 years were 48.6% and 21.3% post-resection; 22.4% and 8.3% post-biopsy. Hazard ratio for all-cause death for low vs. high volume centres was 1.618 in first 0.7 year post-resection (*p* < 0.0001) and 1.411 in first 0.8 year post-biopsy (*p* = 0.0046).

**Conclusion:**

While 30-day postoperative mortality rates were above predefined targets, no association between hospital volume and mortality was found. However, survival probabilities demonstrated benefits from treatment in higher volume centres, particularly in the initial months post-surgery. These variations highlight the need for continuous improvement in neuro-oncological practice and should stimulate reflection on the neuro-oncological care organisation in Belgium.

**Supplementary Information:**

The online version contains supplementary material available at 10.1007/s11060-024-04776-2.

## Introduction

In glioblastoma (GBM) treatment, maximal safe surgical resection followed by chemoradiotherapy according to Stupp’s protocol is considered the standard of care [1, 2]. However, treatment decisions, including surgical options, may vary across hospitals and physicians, resulting in treatment variations and possibly outcome variations. If an attempt at complete resection of a GBM results in functional deficits, it not only affects the quality of life but also jeopardizes the chances for timely adjuvant treatment and, consequently, the prognosis [3]. Survival outcomes after surgery for GBM vary across different published reports [4–16]. Also, among different hospitals within a nation, treatment patterns but also mortality and survival may differ [4, 17–19].

So far, correlations between the quality of care or outcome for patients with GBM and the volume of the centre where they are diagnosed, treated and followed-up have not yet been explored in Belgium.

The objective of this nationwide study was to analyse interhospital variability in 30-day postoperative mortality and the 1- and 2- year survival for GBM patients diagnosed between 2016 and 2019 in Belgium. GBM patients who underwent a (stereotactic) biopsy or a surgical resection were investigated separately. Associations between case load of the centre on the one hand, and survival or mortality on the other hand, were explored, taking into account prognostic factors (sex, age, WHO performance status, comorbidities and other tumours).

## Materials and methods

### Data collection and study cohort

For this study, all GBM patients (ICD-O-3: 9440/3, 9441/3, 9442/3, 9445/3 - C71) diagnosed between 2016 and 2019 were identified in the database of the Belgian Cancer Registry (BCR). (Details in Supplement 1)

### Hospital allocation

Benchmarking for mortality and for 1-/2-year survival was based on the centre performing the biopsy or the surgical resection. Patients who underwent a resection after a biopsy were incorporated for postoperative mortality in both groups. Targets for 30-day postoperative mortality were defined by consensus among a multidisciplinary panel, consisting of specialists with known clinical experience and/or previous scientific contributions in neuro-oncology from both academic and non-academic centres, as < 3% after resection and < 5% after biopsy. Both international target reference values and results were considered during this process. The surgical case load of a hospital, enabling mortality and survival analysis in relation to hospital exposure to glioma, was defined as the number of unique patients undergoing at least 1 intervention (biopsy or resection) for a primary or recurrent glioma during the reference period (2016–2019) in the hospital. This means that only 1 intervention per patient per centre was counted and patients operated (biopsy or resection) at different centres were counted once at each centre. The hospital volume (category) was expressed as a total over the 4-year study period.

### Statistical analysis for 30-day postoperative mortality

The association between hospital volume and mortality within 30 days after surgical resection or biopsy was investigated using logistic regression models. Details are in Supplement 2.

### Statistical analysis for survival analysis

The hazard for all-cause mortality since resection or biopsy was modelled using Cox proportional hazard (PH) models. First the PH assumption was evaluated separately for every case-mix variable and a categorical hospital volume variable. The same case-mix variables as for the 30-day mortality analysis were included. Cumulative hazard curves and comparison of the fitted survival lines from a proportional hazard model to the observed Kaplan-Meier curves were used to assess the PH assumption. Further details in Supplement 3.

## Results

### Cohort description and centre assignment

**Fig. 1:**
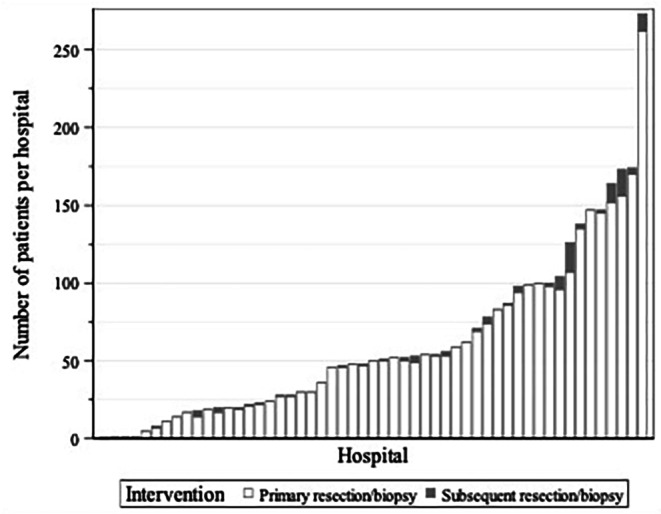
Surgical case load for glioma patients per hospital in Belgium Each bar represents a different hospital (*n* = 53). Hospital case load is defined as the number of patients undergoing at least 1 intervention (biopsy or resection) for a primary or for recurrent glioma (ICD-O-3: 938–945) during the reference period (2016–2019) for each hospital. This means that only 1 intervention per patient is counted per centre and patients treated at different centres are counted once at each centre. White: first interventions (biopsy or resection). Grey: subsequent interventions (biopsy or resection) following one or more interventions in another centre

A total of 2269 newly diagnosed GBM patients were identified in the BCR database between 2016 and 2019. Surgical resection was performed in 1665 cases, of which 159 received both a biopsy and a surgical resection. A (stereotactic) biopsy was performed in 662 patients (followed or not by a surgical resection). In 99 cases (4.4%), the diagnosis of GBM was based solely on imaging. Surgical procedures and tumour characteristics can be found in Table 1 and Supplement 4. The distribution of the hospitals’ surgical case load is illustrated in Fig. 1.

**Table 1:** Summary of applied surgical procedures for glioblastoma in Belgium in 2016–2019

biopsy	503 (22.2%)	662	total biopsy
biopsy + resection	159 (7.0%)		
		1665	total resection
resection	1508* (66.4%)		
no surgery or biopsy	99 (4.4%)		
**total GBM cases from BCR**	**2269**		

*For 2 patients the centre of surgical resection is unknown

### 30-day mortality after resection

**Fig. 2:**
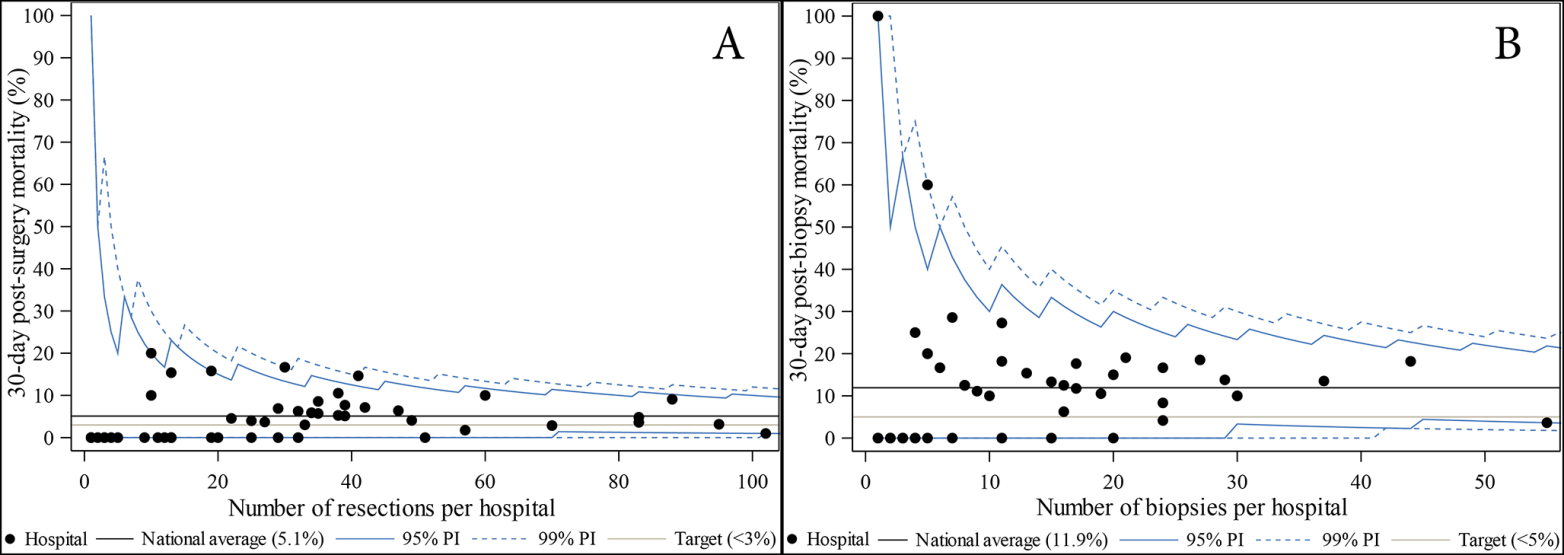
Funnel plots of proportion of GBM patients who died within 30 days of (**A**) a diagnostic biopsy or (**B**) a surgical resection. Although all centres perform within the random variability around the population mean, different low- and medium-volume centres have a post-biopsy mortality rate of around 20%, which contributes to the high national average. (The hospital volumes in these funnel plots reflect the number of observations available per hospital for the regression analysis and are smaller than the “volumes” given in the previous graph. Each patient is assigned to only one hospital.)

At population level, the 30-day postoperative mortality after surgical resection was 5.1% (predefined target < 3%). The 30-day post-resection mortality by case-mix and hospital surgical volume is presented in Supplement 5. A funnel plot illustrates the observed variability among the different centres in Fig. 2A. Although 24 centres had a 30-day post-resection mortality above the predefined target, no centres had a mortality beyond the random variability around the population mean. Results seem comparable in historical comparison periods (2012–2015: 4.4%; 2008–2011: 5.3%) (Supplement 6). (Unadjusted) mortality differs by age group (*p* = 0.0006) reaching 9.3% for the oldest age group (≥ 75y) and by different WHO-status (*p* < 0.0001) reaching 22.3% for patients presenting with WHO-score ≥ 3. Significant higher mortality rates were also seen in patients with cardiovascular disease (6.5%; *p* = 0.0143) or diabetes (10.7%; *p* = 0.0001). The categorical volume variable for the post-surgery models grouped hospitals with < 57 (low), 58–138 (medium) and > 138 patients (high). Adjusted Odds ratio (OR) for 30-day post-resection mortality by hospital volume groups can be found in Table 2. No statistically significant difference in post-surgical mortality versus surgical case load of the hospital was identified (*p* = 0.170). Also, the adjusted OR, using hospital volume as a continuous variable, failed to achieve statistical significance (OR = 0.997; 95% CI [0.992, 1.002]; *p* = 0.3013).

**Table 2:** Adjusted OR’s for hospital volume groups for 30-day post-resection and post-biopsy mortality

**Effect**	**Odds ratio**	**95% CI**	***p*** **-value**	**Type III** ***p*** **-value**
***POST RESECTION***				
***Hospital volume group***				0.5328
< 57 vs. > 138	1.062	[0.465, 2.423]	0.8847	
57–138 vs. > 138	1.496	[0.634, 3.530]	0.3510	
**Effect**	**Odds ratio**	**95% CI**	***p*** **-value**	**Type III** ***p*** **-value**
***POST BIOSPY***				
***Hospital volume group***				0.4169
< 57 vs. > 104	1.291	[0.636, 2.622]	0.4709	
57–104 vs. > 104	1.612	[0.784, 3.316]	0.1887	

### 30-day mortality after biopsy

At population level, the 30-day postoperative mortality after biopsy was 11.9%, being above the predefined target (< 5%). The 30-day post-biopsy mortality by case-mix and hospital volume is presented in Supplement 7. A funnel plot illustrates the observed variability among the different centres (Fig. 2B). Although 27 centres had a 30-day post-biopsy mortality above the predefined target, no centres had a mortality beyond the random variability around the population mean. Results were comparable in historical comparison periods (2012–2015: 11.2%; 2008–2011: 9.5%)(Supplement 6). (Unadjusted) mortality differs by age group (*p* = 0.0134) reaching 17.2% for the oldest age group (≥ 75y) and by different WHO-status (*p* < 0.0001) reaching 25.4% for patients presenting with WHO-score ≥ 3. Again, significantly higher mortality rates were found in patients with cardiovascular disease (14.7%; *p* = 0.0190) or diabetes (19.4%; *p* = 0.0173). The categorical volume variable for the post-biopsy models grouped hospitals with < 57 (low), 57–104 (medium) and > 104 patients (high). Adjusted Odds ratio (OR) for 30-day post-biopsy mortality by hospital volume groups are presented in Table 2. No statistically significant difference in post-biopsy mortality versus surgical case load of the hospital was identified (*p* = 0.7527). The adjusted OR, using hospital volume as a continuous variable, did not reach statistical significance (OR = 0.996; 95% CI [0.992, 1.000]; *p* = 0.0579).

### Observed 1-/2- year survival probability after resection

**Fig. 3:**
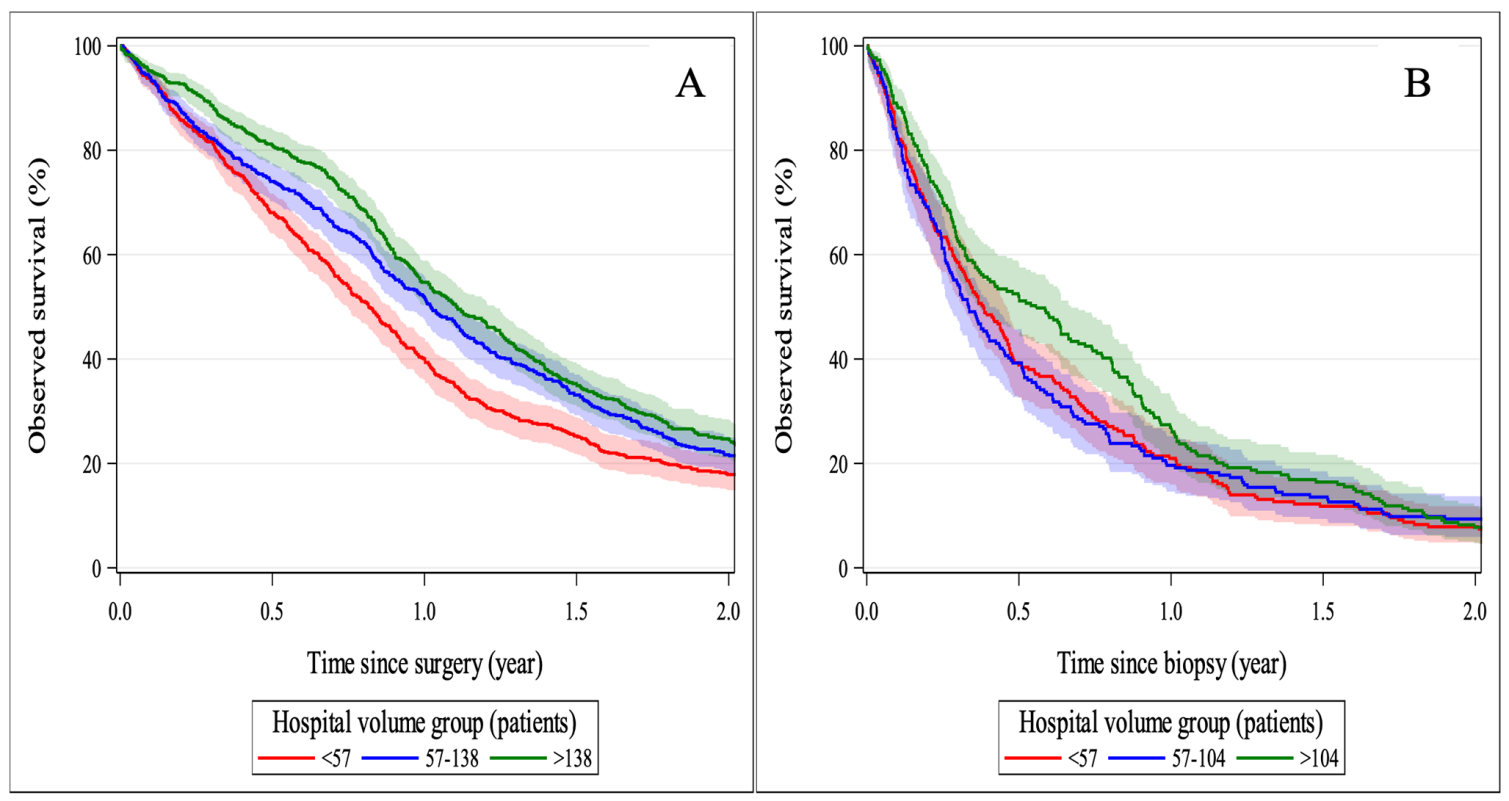
Kaplan Meier curves for the unadjusted observed survival in glioblastoma, stratified by hospital volume groups, after (**A**) surgical resection (*p* < 0.001) and (**B**) biopsy (*p* = 0.1599)

The unadjusted 1-year observed survival (OS) for glioblastoma patients after surgical resection was 48.6%, while the unadjusted 2-year OS is 21.3% (Supplement 8). The unadjusted 1-year OS after resection differed by age group (*p* < 0.0001), dropping to 21.1% for the oldest age group (≥ 75y) and by WHO status at presentation (*p* < 0.0001), declining to 35.9% for patients with WHO-status 2 and to 29.8% for those with WHO-status ≥ 3. Also, in patients with comorbidities 1-year OS was reduced: diabetes: 33.5%; *p* < 0.0001; respiratory disease 36.0%; *p* = 0.0013; cardiovascular disease: 37.2%; *p* < 0.0001. Median survival time (MST) for GBM after resection was 1 year. The categorical volume variable for the post-surgery models grouped hospitals with < 57, 57–138 and > 138 patients. Kaplan-Meier curves for the unadjusted observed survival stratified by hospital volume groups can be found in Fig. 3A. Differences in survival probabilities for the 3 volume groups remained at 2 years after resection. Higher hazard ratios for all-cause death after resection for low vs. high volume centres (1.618; *p* < 0.0001) and medium vs. high volume centres (1.426; *p* = 0.0019) were significant in the first 0.7 year (8.4 months) after resection (Table 3). No statistically significant differences in hazard to die remained between the volume groups in the [0.7y, 2.0y] interval since resection. The optimal binary volume threshold determination resulted in contrasting hospitals with fewer than 120 vs. 120 or more patients for the post-surgery analysis.

**Table 3:** Adjusted hazard ratio for all causes of death since resection and biopsy in GBM vs. hospital volume

Effect	Adjusted hazard ratio (since resection)	95% CI	*p*-value
***Hospital volume group***		**Since resection**	
< 57 vs. > 138 (< 0.7 years)	1.618	[1.307, 2.004]	< 0.0001
57–138 vs. > 138 (< 0.7 years)	1.426	[1.140, 1.783]	0.0019
< 57 vs. > 138 (0.7-2 years)	1.046	[0.872, 1.255]	0.6298
57–138 vs. > 138 (0.7-2 years)	0.941	[0.788, 1.123]	0.4994
***Hospital volume group***			
< 120 vs. > = 120 (< 0.7 years)	1.615	[1.339, 1.949]	< 0.0001
< 120 vs. > = 120 (0.7-2 years)	1.068	[0.918, 1.241]	0.3940
**Effect**	**Adjusted hazard ratio**	**95% CI**	*p*-value
***Hospital volume group***		**Since Biopsy**	
< 57 vs. > 104 (< 0.8 years)	1.411	[1.112, 1.790]	0.0046
57–104 vs. > 104 (< 0.8 years)	1.467	[1.156, 1.861]	0.0016
< 57 vs. > 104 (0.8-2 years)	0.841	[0.570, 1.241]	0.3834
57–104 vs. > 104 (0.8-2 years)	0.527	[0.341, 0.814]	0.0039
***Hospital volume group***			
< 100 vs. > = 100 (< 0.8 years)	1.392	[1.147, 1.689]	0.0008
< 100 vs. > = 100 (0.8-2 years)	0.839	[0.597, 1.179]	0.3120

### Observed 1-/2- year survival probability after biopsy

After biopsy, the unadjusted 1-year OS was 22.4%, while the unadjusted 2-year OS was 8.3% (Supplement 9). The unadjusted 1-year OS after biopsy again varied by age group (*p* < 0.0001), dropping to 8.3% for the oldest age group (≥ 75y) and by WHO status at presentation (*p* < 0.0001), decreasing to 13.5% for patients with WHO-status 2 and to 9.0% for those with WHO-status ≥ 3. Similarly, for patients with comorbidities an inferior 1-year survival was found (diabetes: 18.3%; *p* = 0.0153 and cardiovascular disease: 14.4%; *p* < 0.0001). After biopsy, MST was 0.4 years. The categorical volume variable for the post-biopsy models grouped hospitals with < 57, 57–104 and > 104 patients. Kaplan-Meier curves for the unadjusted observed survival stratified by hospital volume groups can be found in Fig. 3B. Two years after biopsy, survival probabilities for the 3 volume groups converged. Also post-biopsy, in the first 0.8 year (9.6 months), significant higher hazard ratios for all-cause death for low vs. high volume centres (1.411; *p* = 0.0046) and medium vs. high volume centres (1.467; *p* = 0.0016) were identified (Table 3). However, in the time period [0.8y, 2.0y] after biopsy, the hazard ratios for the low and medium centres versus the high-volume centres become smaller than 1, which is significant for the medium volume group only (0.527; *p* = 0.0039). The optimal binary volume threshold determination resulted in contrasting hospitals with fewer than 100 vs. 100 or more for the post-biopsy analysis. Kaplan Meier curves for optimized binary volumes can be found in Supplement 10.

## Discussion

The 30-day mortality at population level was 11.9% after biopsy and 5.1% after surgical resection, being above the predefined targets (5% and 3% respectively). Higher rates for 30-day mortality after biopsy and resection were found in elderly patients (≥ 75y) (17.2% and 9.3%) and in patients with worse WHO-status (e.g. WHO-status ≥ 3: 25.4% and 22.3%). International comparison is difficult since most series consider postoperative mortality for both resection and biopsy combined or evaluate different tumour entities, sometimes including meningioma, together. In Spain (2008–2010) for glioblastoma, the 30-day mortality after resection (4.4%) was comparable with the result in this series, but 30-day mortality after biopsy (14.2%) is slightly higher [9]. In Scotland (2021) the 30-day mortality rate for brain/CNS cancer was 5.0% at population level, but figures differ regionally from 2.3 to 9.0%, while in the Netherlands (only GBM) it was 5.2% [4, 20]. In the Danish Neuro-Oncology Registry, post-surgical mortality for all types of glioma ranged from 2% to 4% between 2010 and 2014 (target set at < 5%) [21]. In the US, based on the Nationwide Inpatient Sample, in hospital mortality rate was 2.3% for needle biopsy, 3.2% for lobectomy and 2.7% for other resections [22]. In single centre series in different countries 30-day mortality rates between 1.5% and 10.7% were reported [9, 21, 23–34]. De Witt Hamer concluded that mortality within 30 days is not a useful quality indicator for glioblastoma-related complications. This is probably true if biopsy and resection are combined. If only resection is considered, early mortality in patients with irresectable tumours in whom only a biopsy is taken, will not confound post-resection operative mortality rates [4]. Indeed, mortality after biopsy accounts for both fatal procedural (haemorrhagic) complications and for true rapid disease progression. However, reporting post-biopsy mortality for glioblastoma patients could also reveal aberrant rates indicating poor surgical technique or poor case selection and futile tissue sampling. Anyway, the high mortality rates after biopsy in elderly patients and in those with worse WHO-scores (sometimes presenting with a pathognomonic MRI) highlight the need for careful multidisciplinary consideration during medical decision making in these subgroups. In this series, 99 patients (4.4% of all registered GBM cases) were withhold from tissue sampling and received presumably supportive care after radiological diagnosis. No differences in early mortality (after biopsy and resection) were identified between the 3 volume groups in this study, contrasting the findings in the Netherlands by De Witt Hamer, who demonstrated an association of higher case volume with lower early mortality [4]. In that series, an estimated boundary between higher and lower than average early mortality was found at 45 patients/year [4]. Also, in a retrospective analysis of a population based cohort in the state of New York (2005–2014), not only the 30-day mortality rate after brain tumour resection (not only glioma) was lower when surgery was performed by surgeons with a high annual (*n* ≥ 24) and high cumulative volume (≥ 114 in 5 years), but also a lower rate of complications and reoperations within 30 days [35]. Contrarily, in Spain, no relationship between the number of major complications (including 30-day mortality) and case load of the surgical centres was identified [9]. Similarly, in England, with a larger median hospital case load (*n* = 273 over a 3-year period), suggesting greater centralisation, the 30-day mortality of intracranial neoplasm resection was not related to hospital volume, but to the individual a surgeon’s case load. A doubling of a surgeon’s caseload resulted in a 20% risk reduction. This suggests a need for further sub-specialisation among neurosurgeons, even within already well-concentrated brain surgery care [36].

International comparison of survival at population level for the subgroups of biopsied or resected patients is cumbersome, since most series don’t differentiate survival results. The 1-year observed survival probability since surgical resection (48.6%) is comparable with the result in Denmark after partial resection (est. 50% on Kaplan-Meier plot), but inferior to the result for the group in Denmark after complete resection (est. 70% on Kaplan-Meier plot) [37].

In Western Norway, MST after resection was 13.7 months and after biopsy 8.3 months, both being superior to the results in our cohort (8.3 months and 4.8 months resp.) [38]. The 1-year survival after biopsy (22.4%) was comparable to the result in Denmark (est. 20% on Kaplan-Meier plot), but superior to the Netherlands were 1-year survival after biopsy and chemoradiation was 15% [37, 39]. In Italy, 1-year survival probability after biopsy for the glioblastoma patients being treated with radiotherapy, was superior to the survival probability for all biopsied patients in Belgium (36.8% vs. 22.4%) while the 2-year probabilities were comparable (7.6% vs. 8.3%) [13]. Two-year post-surgical survival (21.3%) was only slightly inferior to the 2-year survival of 26.5% in the Stupp trial, where 84% of patients underwent resection [1].

Taking into account case-mix variability, survival probability was worse in the first 0.7 years after surgical resection (0.8 years after biopsy) in low- (OR: 1.883; *p* < 0.0001) and medium- (1.419; *p* = 0.0019) vs. high-volume centres. This is again in contrast to the findings in the Netherlands, where higher volume was not related to 2-year survival, but this mixed cohort consisted of both biopsied and resected patients [4]. In Finland, all intracranial surgeries are performed in five university hospitals. Patients with newly diagnosed GBM had a 19% higher relative excess risk (RER) when diagnosed and treated in low-volume centre (mean annual case load = 17) than in the high- and medium-volume hospitals (mean annual case load = 54 and 40 resp.) [19]. The optimal binary volume threshold in this series determined at 120 patients/4 years for resection and 100/4 years for biopsy, suggests that GBM patients might have a better outcome when undergoing surgical treatment in a centre performing on average at least 30 glioma surgeries annually.

In a recent survey by the EANS (European Association of Neurosurgical Societies) the majority of respondents agreed that an expert reaches an annual case load of more than 50 consisting of gliomas, meningiomas and metastases [40]. In Belgium, ideas about organisation and centralisation of patients with *rare and complex tumours* were launched based on studies by the Belgian Health Care Knowledge Centre (KCE) [41, 42]. According to the definition of RARECARE, *rare cancers* have an incidence rate less than 6/100.000 persons/year, categorising almost all subtypes of primary brain tumours (including gliomas) as rare [43]. Grade 4 glial tumours, mainly molecular glioblastoma (age-standardized incidence rate (ESR) = 6.72), are just surpassing this threshold [44]. *Complex cancers* are those in specific and difficult anatomical regions and those requiring high technological or expensive infrastructure [41]. Although subjective, the infrastructure and technologies required for state-of-the-art neuro-oncological surgeries (e.g., neuronavigation, neuro-monitoring, awake brain mapping, fluorescence-guided resection,…) could meet this requirement. These findings and considerations seem to contrast with the current dispersion with 53 centres providing surgical care for GBM (Fig. 1). (In 2019, Belgium had 103 hospitals, 7 of which were university hospitals [45]). The evaluation of the use of the aforementioned technologies and the search for an association with mortality and/or survival in GBM was beyond the scope of this study. Previously, these surgical aids were not considered standalone quality (structure) indicators by our group, but rather tools in achieving maximal safe resection [46].

This population-based study shows for the first time an association of hospital volume and survival after resection/biopsy in patients with a glioblastoma. While in this study expertise is defined by a centre’s surgical case load, it is also crucial to recognize that the management of GBM is multidisciplinary, involving neuropathologists, neuroradiologists, radiation oncologists, medical oncologists, and paramedical staff. As a result, centres with higher surgical load typically also handle a higher volume of cases in the other involved specialties, fostering a group expertise, which might contribute to the improved outcome observed in these centres. This is supported by the work of Haque et al. showing that patients with histologically confirmed GBM receiving chemoradiation at high volume facilities have an improved survival [47]. Moreover, decisions to withhold GBM patients from surgery, adjuvant treatment or, any treatment at all, should ideally be made by a multidisciplinary expert team, of course in consent with the patients and their proxies.

This study also harbours limitations. First, outcome indicators were evaluated based on the WHO-2016 classification, still incorporating the prognostically more favourable IDH-mutant *glioblastoma* (now grade 4 IDH-mutant astrocytoma). However, the limited number of cases (*n* = 16; 0.5%) likely had minimal impact on the survival. Survival and incidence for molecularly defined glial tumours have been previously published by our group, demonstrating a MST for IDH-wildtype glioblastoma of 9.3 months [44]. Another limitation is that true postoperative mortality cannot be distinguished from mortality due to rapid disease progression, as the exact cause of death is unknown in this cohort. Next, a minor limitation is the exclusion of patients who died at incidence date, potentially following a resection or biopsy (*n* = 2). However, in these rare instances, it is challenging to determine whether death resulted from (surgical) complications or was attributable to the possible moribund status upon presentation of a (probably haemorrhagic) GBM. Finally, possible variations in upfront excluding poor-prognosis patients with a radiological suspicion for GBM (4.4% in this series) from a diagnostic biopsy or a resection might influence final outcome data in the pathologically proven GBM populations.

## Conclusion

Reporting postoperative mortality rates and survival for GBM patients is crucial for understanding outcome, at the national and hospital level. High mortality rates after biopsy in elderly patients and in patients with worse WHO-scores highlight the need for careful consideration during medical decision making in these subgroups. Although in this nationwide analysis no correlation between hospital volume and 30-day postoperative mortality for both biopsied and resected GBM patients could be detected, the overall mortality rates being above the predefined targets, should prompt neuro-oncological surgeons to continuously improve their practice and consider carefully therapeutic/diagnostic procedures in elderly and frail patients. Both 1- and 2-year post-biopsy and post-resection survival are in line with international findings. However, significant differences related to hospital case load should open reflections on the organisation of neuro-oncological care in Belgium.

## Electronic supplementary material

Below is the link to the electronic supplementary material.

Supplementary Material 1

## Data Availability

The cancer cohort data used and analysed during the study are available via the Belgian Cancer Registry (BCR) upon reasonable request. The pseudonymized data can be provided within the secured environment of the BCR after having been guaranteed that the applicable GDPR regulations are applied.
