# Supplementary material for: Association of hospital volume with survival but not with postoperative mortality in glioblastoma patients in Belgium
Source: J Neurooncol. 2024 Aug 2;170(1):79–87. doi: 10.1007/s11060-024-04776-2 (PMC11447078; doi:10.1007/s11060-024-04776-2)
Supplement: Supplementary file 1 — Supplementary Material 1 [file 11060_2024_4776_MOESM1_ESM.docx]

Supplement

*Supplement 1: Data collection and study cohort*

In Belgium, cancer registration is compulsory for both hospitals and laboratories for pathological anatomy, as described extensively in previous publications [21, 22]. Using the National Social Security Number (NSSN) as unique patient identifier, the Belgian Cancer Registry (BCR) database can be linked with the database of the Intermutualistic Agency (IMA), providing access to all cancer-related diagnostic and therapeutic procedures reimbursed by the compulsory national health insurance, and with the Crossroads Bank for Social Security, to retrieve vital status information, including in case, date of death. For this study, all GBM patients (ICD-O-3: 9440/3, 9441/3, 9442/3, 9445/3 - C71) diagnosed between 2016 and 2019 meeting the following inclusion criteria were identified in the BCR database: patients of 18 years or older, with official residence in Belgium at time of diagnosis and a known NSSN. Exclusion criteria were: patients without cancer-related diagnostic or therapeutic billing codes available within the time frame 1 month before until 3 months after the incidence date, patients for whom the incidence date was the same as the date of death and patients lost to follow-up at incidence date (i.e., no vital status tracking available).

*Supplement 2: Statistical analysis for 30-day postoperative mortality*

The association between hospital volume and mortality within 30 days after surgical resection or biopsy was investigated using logistic regression models. To account for the correlation in the outcome of patients assigned to the same hospital, a random effect for hospital was added to every model. Hospital volume was converted into a categorical variable based on the terciles, resulting in similar patient representation (in absolute numbers) within each of the 3 volume groups. For all analyses, both unadjusted and adjusted (accounting for the case-mix), odds ratios are reported. To correct for the case-mix, the following variables were included: sex, age at diagnosis (<50, 50-74 and ≥75), WHO performance score at time of diagnosis, comorbidities (diabetes, respiratory disease and cardiovascular disease) and occurrence of other tumours between five years before and two years after incidence date of the GBM. Two-way interactions were tested at a 0.01 significance level using a backwards elimination selection procedure. The model fit was assessed through the Hosmer-Lemeshow test, inspection of the residuals and by verifying whether the deviance to the degrees of freedom stayed below 2.

*Supplement 3: Statistical analysis for survival analysis*

In case the proportional hazard assumption was violated, a piece-wise proportional model is applied. For such a model, the survival time is divided into subsequent time intervals in which proportional hazards can be assumed, for example [0, 0.5] and [0.5, 2.0] year. The location of the final timepoint is optimised by comparing piece-wise proportional models with different timepoints using the AIC (the model with the lowest AIC is selected). The case-mix variables and their potential non-proportionality terms were then joined into a single model, after which two-way interactions among case-mix variables were tested at a 0.01 significance level using a backwards elimination selection procedure. This resulted in the final case-mix model to which the continuous or categorical hospital volume variable was added. Furthermore, an optimal binary volume threshold was explored by comparing models with a threshold at different volume values on the basis of the AIC. For the categorical hospital volume approach, models at 2 years only are sufficient as the non-proportional hazard modelling already takes potential time evolutions over the time interval [0, 2.0] years into account. Finally, the model fit is evaluated by inspection of the Schoenfeld and the Cox-Snell residuals. A significance level of 0.05 was applied in the reporting of the statistical analysis results. All analyses were performed with SAS version 9.4 (SAS Institute, Cary, NC).

Supplement 4. Distribution of WHO-2016 defined glioblastoma

| **Glioblastoma (N=2 269)** | | | |
| --- | --- | --- | --- |
| 9440/3 | glioblastoma | 2 180 | 96.0% |
| 9441/3 | giant cell glioblastoma | 37 | 1.6% |
| 9442/3 | gliosarcoma | 36 | 1.6% |
| 9445/3 | IDH-mutant  glioblastoma* | 16 | 0.7% |

* According to WHO-2021: grade 4 IDH-mutant astrocytoma

| Supplement 5. 30-day post-resection mortality in GBM by case-mix and hospital volume. |
| --- |
|  |
|  |
| \| **Variable** \| **N** \| **Ndeaths** \| **probability (%)** \| **95% CI** \| **p-value** \| \| --- \| --- \| --- \| --- \| --- \| --- \| \| ***Overall*** \| 1,665 \| 85 \| 5.1 \| [4.1, 6.3] \|  \| \| ***Hospital volume group (patients)*** \|  \|  \|  \|  \| 0.4024 \| \| <57 \| 582 \| 33 \| 5.7 \| [3.9, 7.9] \|  \| \| 57-138 \| 562 \| 31 \| 5.5 \| [3.8, 7.7] \|  \| \| >138 \| 521 \| 21 \| 4.0 \| [2.5, 6.1] \|  \| \| ***Sex*** \|  \|  \|  \|  \| 0.3278 \| \| Male \| 1,024 \| 48 \| 4.7 \| [3.5, 6.2] \|  \| \| Female \| 641 \| 37 \| 5.8 \| [4.1, 7.9] \|  \| \| ***Age at diagnosis (years)*** \|  \|  \|  \|  \| 0.0006 \| \| <50 \| 216 \| 8 \| 3.7 \| [1.6, 7.2] \|  \| \| 50-74 \| 1,127 \| 47 \| 4.2 \| [3.1, 5.5] \|  \| \| 75+ \| 322 \| 30 \| 9.3 \| [6.4, 13.0] \|  \| \| ***Cardiovascular disease*** \|  \|  \|  \|  \| 0.0143 \| \| 0 \| 881 \| 34 \| 3.9 \| [2.7, 5.4] \|  \| \| 1 \| 784 \| 51 \| 6.5 \| [4.9, 8.5] \|  \| \| ***Diabetes*** \|  \|  \|  \|  \| 0.0001 \| \| 0 \| 1,459 \| 63 \| 4.3 \| [3.3, 5.5] \|  \| \| 1 \| 206 \| 22 \| 10.7 \| [6.8, 15.7] \|  \| \| ***Respiratory disease*** \|  \|  \|  \|  \| 0.6591 \| \| 0 \| 1,526 \| 79 \| 5.2 \| [4.1, 6.4] \|  \| \| 1 \| 139 \| 6 \| 4.3 \| [1.6, 9.2] \|  \| \| ***WHO performance status*** \|  \|  \|  \|  \| <.0001 \| \| 0 \| 82 \| 3 \| 3.7 \| [0.8, 10.3] \|  \| \| 1 \| 1,097 \| 21 \| 1.9 \| [1.2, 2.9] \|  \| \| 2 \| 245 \| 26 \| 10.6 \| [7.1, 15.2] \|  \| \| 3+ \| 94 \| 21 \| 22.3 \| [14.4, 32.1] \|  \| \| Missing \| 147 \| 14 \| 9.5 \| [5.3, 15.5] \|  \| \| ***Multiple tumours -5/+2 years*** \|  \|  \|  \|  \| 0.9750 \| \| 0 \| 1,487 \| 76 \| 5.1 \| [4.0, 6.4] \|  \| \| 1 \| 178 \| 9 \| 5.1 \| [2.3, 9.4] \|  \|   ******* *Identification of comorbidities (diabetes, respiratory disease and cardiovascular disease) is based on medication use in the year prior to the glioma diagnosis.*  *** Multiple tumours refer to the presence of additional tumours within a timeframe of 5 years before to maximum 2 years after the incidence date of the glioma diagnosis.* |
|  |
|  |

Supplement 6: 30-day mortality rates after biopsy and resection in glioblastoma in historical comparison period.

| **Glioblastoma who received a surgical resection** | | | |  |
| --- | --- | --- | --- | --- |
|  | **Denominator** | **Numerator** | **Proportion [95%CI] (%)** |  |
| **Study period (2016-2019)** | 1 667* | 85 | 5.1 [4.1, 6.3] |  |
| **Comparison period 1 (2012-2015)** | 1 484 | 66 | 4.4 [3.5, 5.6] |  |
| **Comparison period 2 (2008-2011)** | 1 421 | 75 | 5.3 [4.2, 6.6] |  |
| **Glioblastoma who received a diagnostic biopsy** | | | |  |
|  | **Denominator** | **Numerator** | **Proportion [95%CI] (%)** |  |
| **Study period (2016-2019)** | 662 | 79 | 11.9 [9.6, 14.6] |  |
| **Comparison period 1 (2012-2015)** | 625 | 70 | 11.2 [9.0, 13.9] |  |
| **Comparison period 2 (2008-2011)** | 629 | 60 | 9.5 [7.5, 12.1] |  |

* 2 excluded because centre of resection unknown

Supplement 7. 30-day post-biopsy mortality in GBM by case-mix and hospital volume.

| **Variable** | **N** | **Ndeaths** | **probability (%)** | **95% CI** | **p-value** |
| --- | --- | --- | --- | --- | --- |
| ***Overall*** | 662 | 79 | 11.9 | [9.6, 14.6] |  |
| ***Hospital group (patients)*** |  |  |  |  | 0.3589 |
| <57 | 229 | 28 | 12.2 | [8.3, 17.2] |  |
| 57-104 | 214 | 30 | 14.0 | [9.7, 19.4] |  |
| >104 | 219 | 21 | 9.6 | [6.0, 14.3] |  |
| ***Sex*** |  |  |  |  | 0.7586 |
| Male | 396 | 46 | 11.6 | [8.6, 15.2] |  |
| Female | 266 | 33 | 12.4 | [8.7, 17.0] |  |
| ***Age at diagnosis (years)*** |  |  |  |  | 0.0134 |
| <50 | 65 | 3 | 4.6 | [1.0, 12.9] |  |
| 50-74 | 417 | 45 | 10.8 | [8.0, 14.2] |  |
| 75+ | 180 | 31 | 17.2 | [12.0, 23.5] |  |
| ***Cardiovascular disease*** |  |  |  |  | 0.0190 |
| 0 | 308 | 27 | 8.8 | [5.9, 12.5] |  |
| 1 | 354 | 52 | 14.7 | [11.2, 18.8] |  |
| ***Diabetes*** |  |  |  |  | 0.0173 |
| 0 | 569 | 61 | 10.7 | [8.3, 13.6] |  |
| 1 | 93 | 18 | 19.4 | [11.9, 28.9] |  |
| ***Respiratory disease*** |  |  |  |  | 0.6865 |
| 0 | 603 | 71 | 11.8 | [9.3, 14.6] |  |
| 1 | 59 | 8 | 13.6 | [6.0, 25.0] |  |
| ***WHO performance status*** |  |  |  |  | <.0001 |
| <2 | 385 | 25 | 6.5 | [4.2, 9.4] |  |
| 2 | 141 | 25 | 17.7 | [11.8, 25.1] |  |
| 3+ | 67 | 17 | 25.4 | [15.5, 37.5] |  |
| Missing | 69 | 12 | 17.4 | [9.3, 28.4] |  |
| ***Multiple tumours -5/+2 years*** |  |  |  |  | 0.3670 |
| 0 | 602 | 74 | 12.3 | [9.8, 15.2] |  |
| 1 | 60 | 5 | 8.3 | [2.8, 18.4] |  |

******* *Identification of comorbidities (diabetes, respiratory disease and cardiovascular disease) is based on medication is use in the year prior to the glioma diagnosis.*

*** Multiple tumours refer to the presence of additional tumours within a timeframe of 5 years before to maximum 2 years after the incidence date of the glioma diagnosis.*

Supplement 8. 1- and 2-year observed survival estimates after resection in GBM, stratified by case-mix and hospital volume.

|  | | **Unadjusted observed survival since surgery, %** | | | |  | |
| --- | --- | --- | --- | --- | --- | --- | --- |
|  | | **At 1 year** | | **At 2 years** | |  | |
| **Characteristic** | **Initial number at risk** | **estimate** | **95% CI** | **estimate** | **95% CI** | **Median survival (years)** | **p-value** |
| ***Overall*** | 1,665 | 48.6 | [46.2, 51.0] | 21.3 | [19.3, 23.3] | 1.0 |  |
| ***Hospital volume group (patients)*** |  |  |  |  |  |  | <.0001 |
| <57 | 582 | 40.0 | [36.0, 44.0] | 17.9 | [14.9, 21.1] | 0.8 |  |
| 57-138 | 562 | 51.8 | [47.6, 55.8] | 21.7 | [18.3, 25.1] | 1.0 |  |
| >138 | 521 | 54.7 | [50.3, 58.9] | 24.7 | [21.1, 28.5] | 1.1 |  |
| ***Sex*** |  |  |  |  |  |  | 0.9635 |
| Male | 1,024 | 49.8 | [46.7, 52.8] | 20.7 | [18.2, 23.2] | 1.0 |  |
| Female | 641 | 46.6 | [42.7, 50.5] | 22.3 | [19.1, 25.6] | 0.9 |  |
| ***Age at diagnosis (years)*** |  |  |  |  |  |  | <.0001 |
| <50 | 216 | 73.6 | [67.2, 79.0] | 41.9 | [35.2, 48.4] | 1.7 |  |
| 50-74 | 1,127 | 51.6 | [48.7, 54.5] | 21.4 | [19.0, 23.8] | 1.0 |  |
| 75+ | 322 | 21.1 | [16.8, 25.7] | 7.1 | [4.7, 10.3] | 0.5 |  |
| ***WHO PS*** |  |  |  |  |  |  | <.0001 |
| 0 | 82 | 59.8 | [48.3, 69.4] | 22.0 | [13.7, 31.4] | 1.2 |  |
| 1 | 1,097 | 52.1 | [49.1, 55.1] | 22.5 | [20.1, 25.0] | 1.0 |  |
| 2 | 245 | 35.9 | [30.0, 41.9] | 18.4 | [13.8, 23.5] | 0.7 |  |
| 3+ | 94 | 29.8 | [20.9, 39.2] | 10.6 | [5.4, 17.8] | 0.5 |  |
| Missing | 147 | 49.0 | [40.7, 56.8] | 23.5 | [17.0, 30.7] | 1.0 |  |
| ***Diabetes*** |  |  |  |  |  |  | <.0001 |
| 0 | 1,459 | 50.7 | [48.1, 53.3] | 22.8 | [20.7, 25.0] | 1.0 |  |
| 1 | 206 | 33.5 | [27.1, 40.0] | 10.7 | [6.9, 15.3] | 0.7 |  |
| ***Respiratory disease*** |  |  |  |  |  |  | 0.0013 |
| 0 | 1,526 | 49.7 | [47.2, 52.2] | 21.8 | [19.8, 23.9] | 1.0 |  |
| 1 | 139 | 36.0 | [28.1, 43.9] | 15.1 | [9.7, 21.6] | 0.8 |  |
| ***Cardiovascular disease*** |  |  |  |  |  |  | <.0001 |
| 0 | 881 | 58.7 | [55.4, 61.9] | 28.5 | [25.6, 31.5] | 1.2 |  |
| 1 | 784 | 37.2 | [33.9, 40.6] | 13.1 | [10.9, 15.6] | 0.8 |  |
| ***Multiple tumours -5/+2 years*** |  |  |  |  |  |  | 0.0096 |
| 0 | 1,487 | 49.4 | [46.8, 51.9] | 22.2 | [20.1, 24.4] | 1.0 |  |
| 1 | 178 | 42.1 | [34.8, 49.3] | 13.5 | [9.0, 18.9] | 0.9 |  |
| * p-value is based on the log-rank test comparing survival curves up to two years | | | | | | | |

Supplement 9. 1- and 2-year observed survival estimates after biopsy in GBM, stratified by case-mix and hospital volume

|  | | **Unadjusted observed survival since biopsy, %** | | | |  | |
| --- | --- | --- | --- | --- | --- | --- | --- |
|  | | **At 1 year** | | **At 2 years** | |  | |
| **Characteristic** | **Initial number at risk** | **estimate** | **95% CI** | **estimate** | **95% CI** | **Median survival (years)** | **p-value** |
| ***Overall*** | 662 | 22.4 | [19.3, 25.6] | 8.3 | [6.4, 10.6] | 0.4 |  |
| ***Hospital volume group (patients)*** |  |  |  |  |  |  | 0.1599 |
| <57 | 229 | 21.0 | [16.0, 26.4] | 7.9 | [4.8, 11.8] | 0.4 |  |
| 57-104 | 214 | 19.6 | [14.6, 25.2] | 9.3 | [5.9, 13.7] | 0.3 |  |
| >104 | 219 | 26.5 | [20.8, 32.5] | 7.8 | [4.7, 11.8] | 0.6 |  |
| ***Sex*** |  |  |  |  |  |  | 0.2080 |
| Male | 396 | 22.5 | [18.5, 26.7] | 7.8 | [5.5, 10.7] | 0.5 |  |
| Female | 266 | 22.2 | [17.4, 27.3] | 9.0 | [6.0, 12.8] | 0.3 |  |
| ***Age at diagnosis (years)*** |  |  |  |  |  |  | <.0001 |
| <50 | 65 | 56.9 | [44.0, 67.9] | 23.1 | [13.7, 33.8] | 1.1 |  |
| 50-74 | 417 | 23.0 | [19.1, 27.2] | 8.6 | [6.2, 11.6] | 0.5 |  |
| 75+ | 180 | 8.3 | [4.9, 12.9] | 2.2 | [0.7, 5.2] | 0.2 |  |
| ***WHO PS*** |  |  |  |  |  |  | <.0001 |
| <2 | 385 | 28.8 | [24.4, 33.4] | 10.6 | [7.8, 14.0] | 0.5 |  |
| 2 | 141 | 13.5 | [8.5, 19.7] | 5.0 | [2.2, 9.4] | 0.3 |  |
| 3+ | 67 | 9.0 | [3.6, 17.2] | 3.0 | [0.6, 9.3] | 0.1 |  |
| Missing | 69 | 17.4 | [9.6, 27.2] | 7.2 | [2.7, 14.9] | 0.4 |  |
| ***Diabetes*** |  |  |  |  |  |  | 0.0153 |
| 0 | 569 | 23.0 | [19.7, 26.6] | 8.8 | [6.6, 11.3] | 0.4 |  |
| 1 | 93 | 18.3 | [11.2, 26.7] | 5.4 | [2.0, 11.3] | 0.3 |  |
| ***Respiratory disease*** |  |  |  |  |  |  | 0.1306 |
| 0 | 603 | 23.4 | [20.1, 26.8] | 8.6 | [6.6, 11.0] | 0.4 |  |
| 1 | 59 | 11.9 | [5.2, 21.5] | 5.1 | [1.3, 12.8] | 0.3 |  |
| ***Cardiovascular disease*** |  |  |  |  |  |  | <.0001 |
| 0 | 308 | 31.5 | [26.4, 36.7] | 12.7 | [9.2, 16.6] | 0.6 |  |
| 1 | 354 | 14.4 | [11.0, 18.3] | 4.5 | [2.7, 7.0] | 0.3 |  |
| ***Multiple tumours -5/+2 years*** |  |  |  |  |  |  | 0.3965 |
| 0 | 602 | 22.8 | [19.5, 26.2] | 8.5 | [6.4, 10.9] | 0.4 |  |
| 1 | 60 | 18.3 | [9.8, 29.0] | 6.7 | [2.1, 14.8] | 0.3 |  |
| * p-value is based on the log-rank test comparing survival curves up to two years | | | | | | | |

Supplement 10:

Kaplan Meier curves for the unadjusted observed survival, stratified by 2 hospital volume groups after surgical resection (optimal binary volume threshold <120 vs. >= 120)(p < .0001 for [0-0.7y]; p=0.3078 for [0.7-2y]) and biopsy (optimal binary volume threshold <100 vs. >= 100)(p = 0.0021 for [0-0.8y]; p=0.2062 for [0.8-2y])
